# Managing life-altering situations – health needs of families living with recently diagnosed memory disorder

**DOI:** 10.1186/1472-6955-14-S1-S2

**Published:** 2015-10-08

**Authors:** Hanna-Mari Pesonen

**Affiliations:** 1 Faculty of Medicine, University of Oulu, P.O.Box 5000, 90014 Oulu, Finland

## Background

The diagnosis of a memory disorder destabilizes families' lives and affects their orientation towards the future, creating new demands for both the patients and their family caregivers (Steeman et al. 2006) [1]. There is a strong concern both globally and in Europe about establishing services that meet the needs of patients and their family caregivers (World Health Organization 2012). [2] Developing healthcare services that address the needs of those living with a memory disorder requires knowledge produced from service user viewpoints.

## Aim

The aim of this longitudinal study was to describe the mutual processes of managing life after disclosure of a diagnosis of memory disorder from the viewpoints of patients and their family caregivers.

## Methods

Research data were gathered using in-depth interviews (n=40) from patients (n=8) and their family caregivers (n=8). The data were analysed using a constant comparative analysis. Managing life with a memory disorder produces mutual processes in families that contain both positive and negative factors. Family members collaborate to manage their altering life by acknowledging available qualities and resources, seeking meaningful social support and living for today.

## Results

The results call for multi-component and coordinated family-centered care and rehabilitation interventions that strengthen the individuals' and the families' resources, foster hope and empower families to live a meaningful life with the memory disorder. These factors are needed already in the early phases of the condition. Improving families' access to timely and tailored healthcare services can have positive effects on their health.

## References

[B1] Steeman E de Casterlé BD Godderis J Grypdonck M Living with early-stage dementia: a review of qualitative studies Journal of Advanced Nursing 2006 54 6 722 738 16796664 10.1111/j.1365-2648.2006.03874.x

[B2] World Health Organization Dementia: a public health priority 2012

